# Lights for aging: Can photobiomodulation restore functionality in the cerebral networks of aged individuals?

**DOI:** 10.4103/NRR.NRR-D-25-00633

**Published:** 2025-10-30

**Authors:** Marjorie Dole, John Mitrofanis

**Affiliations:** 1 University Grenoble Alpes, Fonds Clinatec, Grenoble, France; 2 University College London, Institute of Ophthalmology, London, UK; 3 University of Turin, Clinical and Biological Sciences, Turin, Italy

**Keywords:** aging, functional connectivity, mitochondria, near infrared, neural regeneration, photobiomodulation, red light

## Abstract

Of all our organs, the brain is particularly prone to aging. The neurons become increasingly more dysfunctional with age, leading to less efficient patterns of functional connectivity, not only within, but also between the different large-scale resting-state networks. Further, the aged brain shows weaker, less efficient patterns of activation when undertaking a particular task, such as a motor movement or recalling a memory. Quite remarkably, however, compensatory mechanisms do develop, where other brain regions are recruited to help perform the tasks. There is, however, much variability across different individuals in the ability to recruit such compensatory processes, and this has been referred to as cognitive reserve or resilience. In addition to these neuronal dysfunctions, the aged brain suffers from widespread inflammation. In terms of options to slow the aging process, there is nothing specific on offer, except for some recommendations to live a healthier life (e.g., change in diet, more exercise, and cognitive training). In this narrative review, we explore the idea that the use of red and near infrared light, referred to often as photobiomodulation, can slow neuronal wear and tear, reduce inflammation, enhance the overall function of the cerebral networks, and improve the quality of life in aged individuals. We hypothesize that the application of these specific wavelengths of light has the potential to improve aged-induced cell dysfunction and death, leading to a restoration of more functional patterns of activity across the aged brain and reducing the risk of developing a neurological disorder such as stroke or neurodegenerative disease.

## Introduction

The world is getting older. The proportion of aged individuals, those older than 65 years, within the general population is increasing steadily. It has been estimated by the World Health Organization that by 2050, the number of individuals with 60 years or more will reach over 2 billion, double the number of children under 5. These statistics raise some serious issues for nearly all sectors of our society; in particular, there will be a greater need for services associated with the housing and health care of this ever-growing elderly population (Peters, 2006; López‐Otín et al., 2013).

Aging is a normal physiological process that happens to all of us. All our body organs suffer from the aging process; the intrinsic function of cells and their vascularization become less efficient, compromising the normal function of the organ. The brain suffers from aging, and this leads to a higher incidence of pathology, such as stroke and/or neurodegenerative disease (Peters, 2006; López‐Otín et al., 2013). Over the past decade, extensive efforts have been made in the neuroimaging and clinical communities to disentangle the numerous factors playing a role in the transition from normal to pathological aging. The objective is to find reliable biomarkers that influence the cognitive and brain morphological trajectories, to propose therapeutical strategies able to reverse the development of neurological/neurodegenerative diseases and delay brain aging. This has been made possible by the development of extended worldwide databases bringing together clinical, structural and functional neuroimaging, and biological data of thousands of patients (for example, the ADNI, Alzheimer’s Disease Neuroimaging Initiative, *n* = 1627, or the UK Biobank) combined with advanced statistical modeling methods such as machine-learning or deep-learning, that allow large-scale and multifactorial analyses (Damoiseaux, 2017; Atkins et al., 2019; Liu et al., 2024). This has recently led in several notions allowing a better characterization of age effects on the brain, and factors influencing brain and cognitive resilience, such as the notion of Brain Age Gap (that is, the deviation between the estimated age of the brain and the real chronological age; Beck et al., 2021; Wrigglesworth et al., 2022; Wittens et al., 2024; Marseglia et al., 2024; Yi et al., 2025), brain maintenance (that is, the preservation of brain structure despite growing age) and cognitive reserve (the preservation of cognitive abilities despite age-related brain changes; Stern et al., 2019; Anatürk et al., 2020; Zhang et al., 2024). Among the therapeutic and preventive options, acting on cardiovascular and metabolic factors and neuroinflammation appears to be crucial in the development of new targets (Marseglia et al., 2024; Na et al., 2025; Tamata et al., 2025). Besides anti-aging drugs such as antidiabetics or Rapamycin (Li et al., 2021), transcranial photobiomodulation appears to be a valid and encouraging option to control these age-related deleterious factors.

In the sections that follow, we will consider the following issues associated with aging: the global features, the cellular changes, alterations in the function of cerebral networks, and the current options available that slow the process. We will then introduce the idea and outline the beneficial outcomes of using red and near infrared light to slow the aging process. We suggest that these wavelengths of light can help alter the aged-induced cellular and functional connectivity changes in the brain, promoting a more functional network system into older brains and reducing the risk of developing a neurological disorder, such as stroke or neurodegenerative disease.

## Search Strategy

Electronic searches of the PubMed database for literature with the key words “ageing, aging, aged, transcranial, photobiomodulation, low level laser therapy, red and near infrared light” were performed. The results were further screened by title and abstract to include articles on animal models and patients that were directly relevant to these key search items. This included publications up to and prior to April 2025. The articles that were not specific to the key words nor directly relevant to the issues considered in this review were not included.

## Aging Process

### Global changes across the aged brain

The major brain functions affected most by aging include perception, memory, and attention. Using memory as an example, both semantic (memory for meanings; e.g., that London is the capital of England) and episodic (memory of where, when, and how things happened; e.g., the occasion of your first date) all decline with age. The age-induced decline in cognitive function is not a uniform process however; processing speed (Salthouse, 1996), working memory (Park et al., 2002), and encoding of episodic memory (Tromp et al., 2015) are among the functions that are, in general, rather severely affected, while other functions, such as semantic memory and language skills (vocabulary and comprehension), tend to remain more stable (Park and Reuter-Lorenz, 2009). Further, although all individuals suffer in some ways from the aging process, some suffer more than others. For instance, overall cognitive decline can be quite severe in some individuals, even in the absence of underlying neurodegenerative disease, while in others, it can be rather minimal (Peters, 2006; López‐Otín et al., 2013; Zanto and Gazzaley, 2019; Aron et al., 2022). This variability has been suggested to rely, at least partly, on differences in “cognitive reserve” of the individual (Stern, 2012; Stern et al., 2023); that is, individual differences in genetics and overall life experiences (e.g., occupation, education and social networking) all have an impact on the ability of the brain to try and offset or limit the deleterious effects of aging (see below; Cabeza, 2002; Davis et al., 2008; Park and Reuter-Lorenz, 2009; Pettigrew and Soldan, 2019).

**Figure 1:**
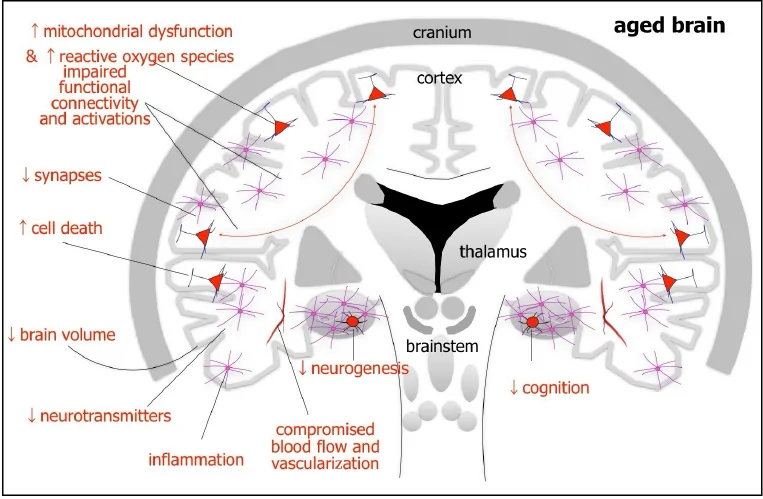
Schematic diagram of frontal section of brain showing the cellular features and the connectivity and activity patterns in the aged brain. The aging process generates many cellular dysfunctions that result in impaired overall brain function.

In terms of morphology, as we age, our brains shrink. One of the primary reasons for this shrinkage is a decrease in the overall number of neurons. This is due to two major factors: (1) a decrease in the generation of new neurons (i.e., neurogenesis), mainly in the brain regions involved in memory and executive functions (i.e., hippocampus and prefrontal cortex), and (2) an increase in cell death. This cell death results in a decrease in neuron number across all areas of the brain, but is most prevalent in the prefrontal areas of the cerebral cortex (**Figure 1**). This region of the brain is involved in a range of higher-order cognitive functions, including logic, decision-making, attention, and working memory (Peters, 2006; López‐Otín et al., 2013; Denburg and Hedgcock, 2015; O’Shea et al., 2016; Zanto and Gazzaley, 2019). Other aged-induced morphological changes in the brain include: enlargement of the ventricles, a global increase in cerebrospinal fluid, together with an increase in neuroinflammatory processes (see below) and de-myelination. This latter feature is reflected in an increase in white matter hyperintensities detected from structural images of the aged brain; these features have been associated with a decrease in the speed of processing information in older subjects (Wiseman et al., 2018).

### Cellular age–related changes: Importance of the mitochondria

**Figure 2:**
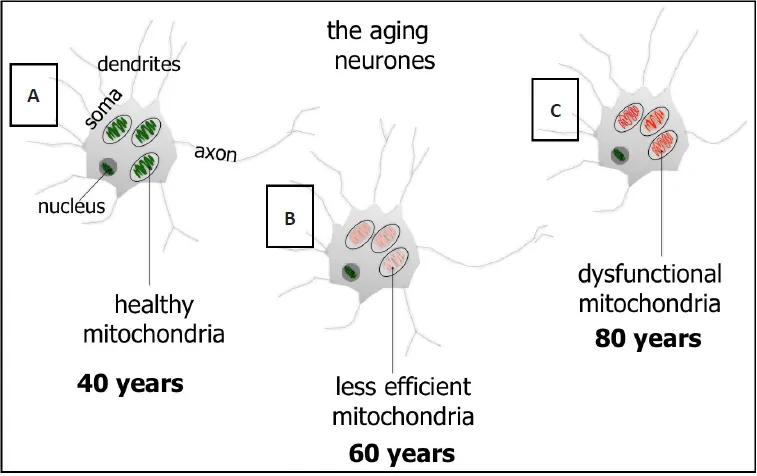
Schematic diagrams depicting the deterioration of the mitochondria during aging. With age, the mitochondria become decreasingly efficient in function (i.e., producing adenosine triphosphate). This process is thought to start at about the age of 60 years.

**Figure 3:**
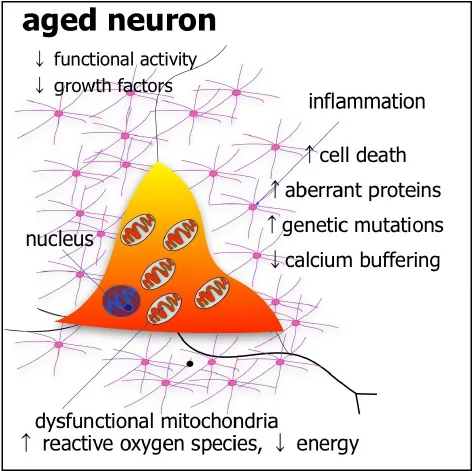
Schematic diagram of the major cellular and molecular changes associated with neurons in the aged brain.

The age-induced cell death is due to a range of factors, including the development of aberrant proteins and several genetic mutations. In more recent years, a dysfunction of key organelles within the cells, called mitochondria, has been shown to be pivotal to the aging process; from approximately 60 years of age, the efficacy of mitochondrial function declines (**Figures 2** and **3**). These organelles are involved in maintaining normal cell function. They are the so-called “engine-rooms” of cells, being the main producers of adenosine triphosphate (ATP) energy, the essential fuel that drives many internal cell functions. They are also the source of reactive oxygen species, which under physiological conditions can act as signaling molecules and maintain homeostasis. Mitochondria have many other key functions, for example, in the buffering of calcium ions, which is critical in the control of cell excitability, neurotransmitter release, and/or gene transcription (**Figure 3**; Peters, 2006; López‐Otín et al., 2013; Denburg and Hedgcock, 2015; O’Shea et al., 2016; Mitrofanis and Jeffery, 2018; Zanto and Gazzaley, 2019).

When mitochondria become dysfunctional, for example, during the aging process, normal cell function is disrupted (**Figures 2** and **3**). First, ATP production decreases and there is a loss of readily available energy to drive intrinsic cell physiology. Second, calcium buffering is impaired, leading to abnormal cell activity and internal damage. Finally, there is an increase in the production of reactive oxygen species, resulting in higher oxidative stress, generating much toxicity that damages the internal cell structure and leads to a high mutation rate of mitochondrial DNA. This oxidative damage leads to severe dysfunction of the cells and ultimately, to their death (Peters, 2006; López‐Otín et al., 2013; Denburg and Hedgcock, 2015; O’Shea et al., 2016; Zanto and Gazzaley, 2019).

Accompanying this cell death, there are a number of associated features across the brain, including: a reduction in the number of synaptic communications, a decrease in the level of neurotransmitters (e.g., catecholamines: dopamine and noradrenaline), reduced levels of brain-derived growth factor (molecule critical for maintaining cell homeostasis and promoting neuronal plasticity important for memory), and alterations of glucose metabolism particularly in frontal and temporal regions (Deery et al., 2023). There is also extensive neuroinflammation across the brain, with the activation of the resident glial cells, astrocytes and microglia (**Figures 1** and **3**; Peters, 2006; López‐Otín et al., 2013; Azam et al., 2021; Jin and Cai, 2023).

In addition, the vasculature across the brain undergoes a progressive change during aging (**Figure 1**). The arteries become stiffer and the pulse becomes ever more distinct, perhaps causing repeated microtrauma that damages the delicate neurons. Blood pressure rises, increasing the incidence of vascular accidents, such as blood clots and endothelial cell damage. Further, alterations in neurovascular coupling have been found in aging brain and this can lead to a diminished supply of nutrients to meet the high metabolic needs of the neurons. Taken all together, the overall homeostasis of the neurons is compromised, generating their dysfunction and ultimate cell death (Peters, 2006; Fabiani et al., 2014; Stone et al., 2015).

### Alterations in brain network function and the way the aged brain fights back with compensations

**Figure 4:**
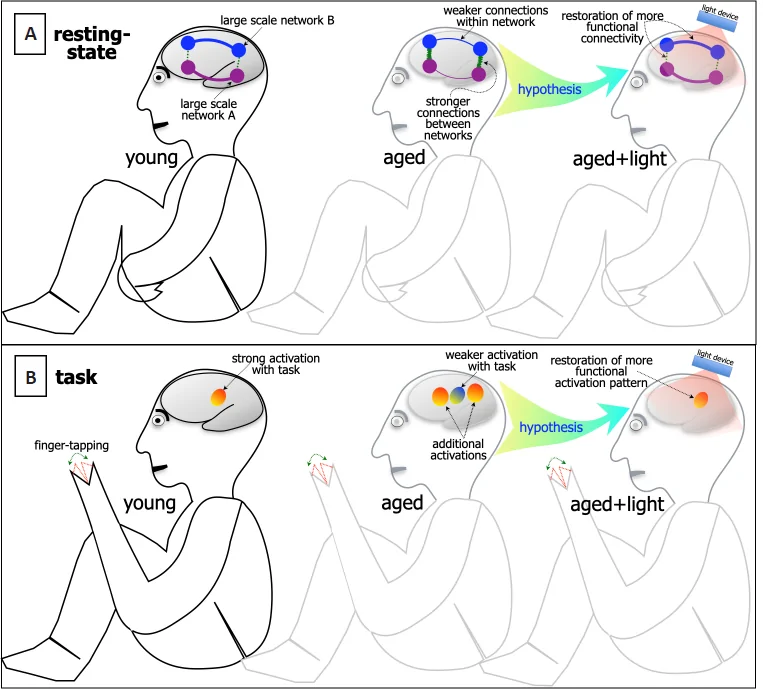
Schematic diagrams of the changes in brain activity in aging during (A) resting-state and (B) task. During resting-state (A), age-related changes include a decrease in connectivity within each large-scale network (e.g., default mode, salience, and sensorimotor) but an increase between them, presumably as compensation. During a task (e.g., finger-tapping movement), there is generally a weaker activation of the specific area associated with that movement (e.g., in M1), but there are additional areas activated as compensation. We hypothesize that light (red and near infrared) treatment will restore more functional patterns of connectivity at rest (A) and activations during task (B), and this treatment will help in the prevention of neurodegenerative disease.

As a result of the growing cellular dysfunction associated with the aging process, the overall function of the brain becomes increasingly compromised. In general, the patterns of activity, at both rest and during a particular task, have been shown to be very different between young and aged brains. For example, when comparing the aged brain to the young one, many studies have reported a clear decrease in functional connectivity within many of the large-scale resting-state networks, namely default mode, salience, and sensorimotor networks, but a concurrent increase in functional connectivity between them. As an example, when performing a particular task, for example a motor movement, the aged brain generally shows a higher and more widespread pattern of activation than the young brain when undertaking the same movement (i.e., across frontal and parietal lobes); in addition, there is generally a weaker activation of the specific motor areas (e.g., within primary motor cortex) associated with generating that movement in the aged brain (**Figure 4A** and **B**). Another example can be found in the decline in memory associated with aging; here, where a particular memory task would involve activation of areas on just one side, with aging, increasingly more areas are activated on the other side of the brain (Mattay et al., 2002; Peters, 2006; Grady, 2013; Martins et al., 2015; Wen et al., 2019; Zonneveld et al., 2019; He et al., 2020; Aron et al., 2022; Deery et al., 2022; Varela-López et al., 2022; Van Ruitenbeek et al., 2023).

These age-related changes in functional connectivity across the resting-state networks and in task-related activations have been related to an adaptation and/or compensation to the growing dysfunction. With the decline in functional efficiency, more networks and areas are recruited to replace those that are no longer functional or accessed easily to help perform the task at hand more efficiently. In other words, the brain fights back and offers some resilience to the aging process (**Figure 4A** and **B**; Mattay et al., 2002; Peters, 2006; Grady, 2013; Martins et al., 2015; Stone et al., 2019; Zonneveld et al., 2019; He et al., 2020; Aron et al., 2022; Varela-López et al., 2022; Van Ruitenbeek et al., 2023).

Aging has been investigated in the framework of graph theory for neural networks (Achard and Bullmore, 2007). According to this theory, an efficient processing of neural information across neural networks requires a balance between functional integration (extensive sharing of information between distant brain areas) and segregation (specialization of brain regions being associated with one or several processes). If there is too much segregation, this will lead to a lack of information transfer; if there is too much integration, then the information flow will become inefficient. Recent findings of graph theory have shown that the aged brain is characterized by an increase in integration and functional connectivity in-between networks (see above), a decreased local and global efficiency and segregation, a decreased modularity, particularly in so-called “rich-club hubs”; these hubs form heavily connected nodes in the brain and are of particular importance for an optimized functioning of the networks (Deery et al., 2022). These graph theory findings are not inconsistent with those reporting the age-induced abnormal recruitment of brain regions and compensatory brain activities observed during several tasks (see above; Cabeza et al., 2002; Davis et al., 2008; Lavanga et al., 2023).

### Current options available that slow the aging process: What can we do?

Although there are some recommendations to slow the aging process, improve mechanisms of cognitive reserve and resilience, as well as prolong our lives, there is nothing too specific. These recommendations generally involve a change in lifestyle, and may include; doing more exercise, eating a healthy diet, lowering alcohol intake and having a consistent source of intellectual stimulation (Peters, 2006; López‐Otín et al., 2013). Pharmacological anti-aging interventions include anti-diabetics (metformin), Rapamycin which reduces the activity of mTOR, a kinase complex, resulting in an immunosuppressive effect, probiotics targeting gut microbiota, or anti-inflammatory drugs. Among these pharmacological treatments, Rapamycin, for example, has gained considerable interest lately because of its potential neuroprotective effects and its positive outcome on cognitive function in animal models of aging (Li et al., 2021; Zheng et al., 2024). In human, whereas this molecule has been shown to improve immune function (Mannick et al., 2018), the effects on cognition and/or brain function/morphology are rather mixed (Kraig et al., 2018; Gonzalez et al., 2021). However, as with other pharmacological interventions, secondary effects have been noted even if no serious adverse even have been recorded (Lee et al., 2024). Hence, any new treatment that can help improve our quality of life in older age, even reducing the neuronal death and inflammation, together with restoring and improving the functional brain connectivity and activity, would be of extreme benefit to the aging population, their families and their careers. Ideally, this treatment should be non-invasive and non-pharmacological, relatively inexpensive, as well as being easy to use with few or no side effects.

## Light

In this context, there is an emerging method, involving the use of red to near infrared light (λ = 600–1300 nm), which may form such a treatment. This treatment, referred to often as photobiomodulation, has been shown to have a major influence on the function and homeostasis of neurons. It relies on using a part of the natural solar spectrum; namely, the red to near infrared wavelengths that are of extreme benefit to the well-being of the cells of our bodies, including those of the brain. Indeed, many experiments in animal models of aging and neurological disease have shown that it is effective in improving neuronal survival and reducing inflammation, and there are some early indications that it can be effective in enhancing cerebral blood flow, functional connectivity, and patterns of activations in humans (see below). It has an impeccable safety record, with little or no evidence of side effects or toxicity on body cells and the devices are easy to use and inexpensive (Hamblin, 2016; Mitrofanis, 2019).

In the sections that follow, the putative mechanisms behind red to near infrared light (henceforth referred to as “light”) are considered, together with its safety record, its penetration capabilities through body tissues, and finally, with what is known of its impact on aging.

### Mechanisms of light

**Figure 5:**
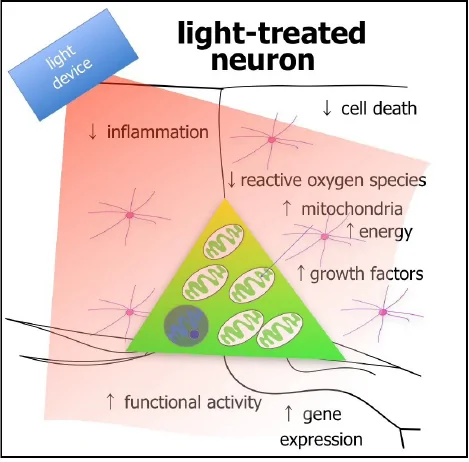
Schematic diagram of the major cellular improvements after light (red and near infrared) treatment on aged neurons. Light treatment seems to turn dysfunctional aged neurons in healthy ones.

**Figure 6:**
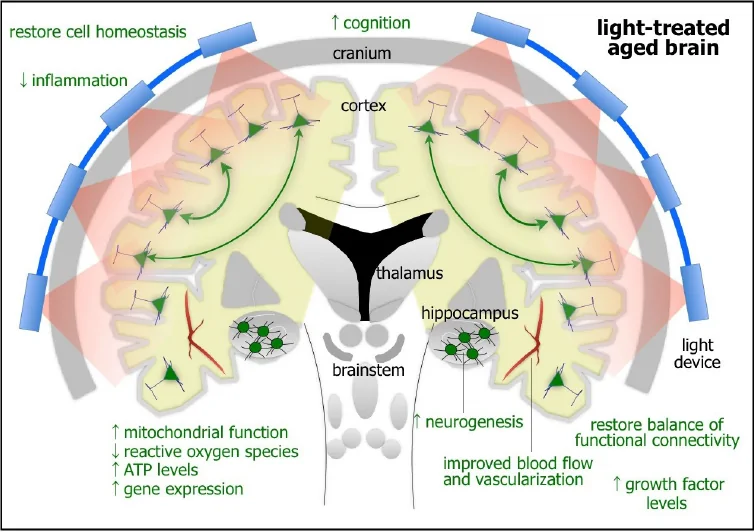
Schematic diagram of frontal section of brain showing the cellular features and the connectivity and activity patterns in the light-treated aged brain. The aging process generates many cellular dysfunctions that result in impaired overall brain function. Light (red and near infrared) treatment has been shown to improve nearly all the dysfunctions and impairments associated with the aged brain.

Many studies over the last seventy years or so have reported that the bulk of the beneficial effects of light centers on its ability to drive mitochondrial activity (**Figures 5** and **6**). The mitochondria have receptors for light across the red to near infrared wavelengths and these receptors such as cytochrome oxidase c and interfacial water trigger the production of more ATP energy. Light can also reduce reactive oxygen species levels and hence any potential stress on the cell under pathological conditions (Hamblin, 2016; Mitrofanis, 2019; Hamblin and Liebert, 2022).

In addition to these short-term energy gains, light also induces more long-term cellular changes by activating the expression of various functional and protective genes; to date, some twenty genes have been associated with light activation (**Figure 5**). In general, light increases the resilience of cells, in particular the neurons (Hamblin, 2016; Mitrofanis, 2019; Stone et al., 2019; Hamblin and Liebert, 2022). Many studies, across a range of neurological disorders, for example Alzheimer’s and Parkinson’s diseases and traumatic brain injury, have reported that light can reduce patterns of neuroinflammation (e.g., Xi et al., 2020), as well as an increase in growth factors (e.g., brain-derived growth factor) that promote a regrowth and repair of neuronal axons and somata (Xuan et al., 2015). Further, light can stimulate the release of nitric oxide into tissues, which can not only improve blood flow in surrounding vessels (by prompting vasodilation), but also improve the function or any damaged local endothelial cells (San Miguel et al., 2019; Colombo et al., 2021). There is also evidence that light prompts angiogenesis by activating the vascular endothelial growth factor pathways (Zhang et al., 2022). These light-induced effects have been considered molecular rather than thermal, mainly because they occur without any large-scale increases in the temperature of the surrounding tissue, particularly when using therapeutic doses (e.g., < 1^o^C; Hamblin, 2016, 2017; Parvin et al., 2023).

It should be noted that all the above-outlined mechanisms depend on light falling directly onto the neurons. This is regarded as the most effective way in which light generates beneficial outcomes. There is, however, another mechanism of action. Light has been shown, quite remarkably, to be beneficial to neuronal survival in the brain even when it is applied to a more distant location (e.g., thigh); that is, when it is not applied directly to the neurons. This indirect stimulation is thought to activate circulating cells or molecules, or even free-floating mitochondria, within the cardiovascular or lymphatic systems that then lead to beneficial effects in the distressed neurons in the brain. The indirect stimulation, although offering neuroprotection, is considered less effective than direct application (Johnstone et al., 2014; Mitrofanis, 2019). Indeed, it has been shown that light treatment can promote a down-regulation of key pro-inflammatory cytokines associated with inflammation, and an up-regulation of anti-inflammatory cytokines in the circulation (e.g., Shamloo et al., 2023). Light has also been recently showed to activate meningeal lymphatic vessels, probably through the release of nitric oxide that dilatate lymphatic vessels, promoting lymphatic clearance of brain macromolecules. This could be of significant interest in various neurological diseases in which toxic waste products accumulate in the brain, such as amyloid-β in Alzheimer’s disease (Semyachkina-Glushkovskaya et al., 2021), proinflammatory cytokines, reactive oxygen species or other toxic proteins in traumatic brain injury or red blood cells in intraventricular hemorrhage (Li et al., 2023). Neuroinflammation have been consistently showed to be reduced after transcranial application of light, by the reduced level of microglia activation in various animal models (dos Santos Cardoso et al., 2022). There is also evidence that light application to the abdomen can influence the survival or neurons and reduce inflammation in the brains of animal models of Alzheimer’s disease, as well as improve the microbiome environment in normal mice and Parkinson’s disease patients.

### Is this light safe?

With over seventy years of experimental and clinical work, there are next to no reports of light having a detrimental effect on body tissue, particularly when being applied at therapeutic doses. Previous studies using external light devices at power intensities ranging from ~1–700 mW/cm^2^, have reported no adverse effect on brain structure and function (Ilic et al., 2006; McCarthy et al., 2010; Quirk and Whelan, 2011; Chung et al., 2012; Tata and Waynant, 2012; Hamblin, 2016). Even when using an internal optical fiber device, implanted surgically within the brain or spinal cord in various animal models, delivering the light directly onto the neural tissue, no toxicity has been reported, even at the higher doses (Moro et al., 2014, 2016; Reinhart et al., 2016; Darlot et al., 2016; Liang et al., 2022). There is one study, however, that has reported some brain damage and adverse behavior from light treatment from an external device in mice (Ilic et al., 2006). This study explored the limits of the treatment and negative outcomes were reached only after the mice were given an exceptionally high-power intensity of 750 mW/cm^2^, approximately one hundred times higher than the dose required to elicit a therapeutic response (< 10 mW/cm^2^; in this animal model). Notwithstanding this single experimental study, the overwhelming body of data indicates that when light is applied at therapeutic doses, and even well above these doses, it has little or no adverse effect on body tissues. In addition, from countless clinical studies over the years, even after long-term use (> 12 months; Boyer et al., 2023), there are very few, if any, reports of any major side effects resulting from light treatment. In a clinical study, Coelho et al. (2025) recently examined the dose-dependent safety aspects in patients with major depressive disorder; they used the SAFTEE-SI scale to investigate systematically the frequency of side effects, of an 808-nm treatment using three different energy densities (low: 60 J/cm², medium: 100 J/cm², high: 180 J/cm²), during four weekly sessions. They also recorded systolic and diastolic blood pressure and found no effect on these measures. They also found no change between baseline and after sham or light on the overall scores. When reported, side effects were rather weak (“stuffy nose,” “apathy,” “diminished mental acuity”; see also Cassano et al., 2019 for comparable results in a small sample size). Importantly, the frequency of side effects did not increase with the increasing dose. A recent study also examined the effect of a single 1064-nm PBM delivered with an energy density of 80 and 180 J/cm² on various parameter, including blood parameters (neuron‐specific enolase and S100β), structural magnetic resonance imaging, cognitive test targeting executive functions, electroencephalogram, and subjective questionnaires (Li et al., 2023). They report no deleterious change in any of these parameters. However, the limited duration of application, with only one 8-minute session in this study makes impossible drawing conclusion about the long-term safety of transcranial light application. When taken all together, light has an excellent safety record, with many government authorities having approved a wide range of devices for home and medical use (Hamblin, 2016, 2017, 2018; Hennessy and Hamblin, 2017), even if more systematic studies are still needed.

### Transcranial application of light: Does it reach the brain and have an effect?

At this point, some comment should be made on the capability of light, delivered by a transcranial device, to penetrate through a series of body tissues, namely hair, skin, bone, meninges, and reach the brain. Notwithstanding some variations — and these can be related to, at least in part, to differences in the wavelengths (red or near infrared), light sources (light emitting devices versus laser), delivery (pulse or continuous), and/or to the different type of tissue used (i.e., formalin-fixed/unfixed cadaver or living) — the distances reported are usually in the range of 20–50 mm (Byrnes et al., 2005; Haeussinger et al., 2011; Moro et al., 2014; Henderson and Morries, 2015; Lapchak et al., 2015; Tedford et al., 2015; Hu et al., 2019). Further, a few Monte Carlo simulations have also been undertaken and these have estimated similar distances, between 20–40 mm (Haeussinger et al., 2011; Pitzschke et al., 2015; Cassano et al., 2019; Yuan et al., 2020; Dole et al., 2024).

In humans, most regions of the cortex lie within 10–15 mm below scalp, seemingly well within reach of light applied transcranially. For our purposes here, the cortex is where much of the dysfunction associated with the aging process occurs (see above) and hence central to our endeavor. Indeed, several of the above-mentioned studies, using either formalin-fixed or fresh tissue or the Monte Carlo simulations, have indicated that both red (~620 nm to ~750 nm) and near infrared (~750 nm to ~1300 nm) light can travel the distances involved from the skin, bone and meninges and reach the depths of the cortex. Further, near infrared light, particularly the wavelengths near 810 nm, has been shown to consistently travel further through the body tissues than red light (see Tedford et al., 2015; Pitzschke et al., 2015; Li et al., 2017; Cassano et al., 2019; Yuan et al., 2020; Dole et al., 2024). It should be noted, however, that many studies have reported massive reductions and loss of power intensity as the light leaves the source and traverses through the cranium and brain parenchyma, being in the order of 90%–98%, even after only a few millimeters of body tissue (DeTaboada et al., 2006; Shaw et al., 2010; Jagdeo et al., 2012; Abdo et al., 2013; Moro et al., 2014); it has been estimated that the reduction is in the vicinity of 65% across each millimeter of brain tissue (Moro et al., 2014). Therefore, it is thought that superficial tissues should be treated by shorter wavelengths, whereas deeper tissues will be treated by longer wavelengths (Selestin-Raja et al., 2024).

The question must be asked then: is there still enough light, delivered from a transcranial device, reaching the cortex in humans to generate an effect? The simple answer to this question is: yes, there is. Many previous studies have provided evidence that light, when applied transcranially, does impact on brain function, and does influence cortico-cortical communication involved in cognition (e.g., memory, attention and executive functions), as well as the major descending pathways that generate movement (Hamblin, 2016; Mitrofanis, 2019; Dole et al., 2023; **Figures 4** and **6**).

To this end, a few studies, using near infrared spectroscopy in young healthy individuals, have shown that transcranially-applied light can have a major metabolic influence in the brain through the activation of cytochrome oxidase c and an increase of hemoglobin oxygenation (Wang et al., 2017; Chan et al., 2019; Urquhart et al., 2020; Saucedo et al., 2021; Lee and Chan, 2023). This would have two overall effects. First, an increased ATP synthesis and oxygen consumption by the stimulated cells that, through neurovascular coupling, increases oxygenated hemoglobin concentration in the stimulated region. Second, cytochrome c activation generates the release of nitric oxide, triggering modifications in the endothelial cells and vascular smooth muscle of the micro-vessels, reflected in changes of endogenic and myogenic rhythms (Truong et al., 2022). These light-induced metabolic changes could be responsible for the following findings: (1) Improvements in cognition, in terms of reaction times or performances in a range of learning and memory retrieval tasks using various cognitive tests (Barrett and Gonzalez-Lima, 2013; Gonzalez-Lima and Barrett, 2014; Blanco et al., 2017a, b; Grover et al., 2017; Jahan et al., 2019); (2) alterations in functional connectivity using functional magnetic resonance imaging (Dmochowski et al., 2020); (3) changes in the resting power spectrum of the different brain waves, increasing α, β and γ band power and decreasing the δ and θ band power using electroencephalogram (Wang et al., 2017, 2019, 2021; Jahan et al., 2019; Shan et al., 2021). The precise ways by which the light-induced metabolic changes result in changes in neural oscillation are not entirely clear, but recent findings have shown that this could be through alterations, or even a reversal, of neurovascular coupling in the stimulated regions (Shahdadian et al., 2024). (4) Alterations in patterns of cortical activation during the performance of a task, more commonly a reduction in activation (but see Bibb et al., 2025). Indeed, several studies have reported light-induced reductions in hemoglobin concentration during the performance of a working memory task using near infrared spectroscopy (Chan et al., 2021; Lee and Chan, 2023), blood-oxygen-level-dependent responses during finger-tapping using functional magnetic resonance imaging (El Khoury et al., 2019), and hand motor-evoked potentials using transcranial magnetic stimulation (Konstantinovic et al., 2013). Such reductions are rather counter-intuitive; one might expect an increase in activity after light treatment. Although the mechanisms are not clear, these reductions in activity could be related to the following scenario. The light-induced metabolic changes generate an increase in the energy and nutrients available to drive the active neurons engaged in a task. Hence, with more readily available energy and nutrients to work with, the active neurons do not need to be as active as they would be undertaking a task under normal, non-light treated, circumstances; their activity would be reduced compared to sham-control.

### What is the optimal light dosage for therapeutic effects?

One of the key issues for light treatment as therapy is dosage. There is so much confusion across the literature generated mainly by differences in choice of wavelength (red or near infrared), delivery method (pulse *versus* continuous), delivery parameters (timing, optical power, total energy, and repetitions), experimental model (*in vivo* or *in vitro*), target cell type (fibroblasts, neurons, or astrocytes) and target tissue (lung, muscle, or brain), as to what the most appropriate dosage to use is. Notwithstanding this confusion, there seems to be a broad range of parameters available, with energies between 1–15 J/cm^2^ and power densities of 10–70 mW/cm^2^ providing the best outcomes in most cases; these values appear to extend a little higher for humans, being in the range of 10–84 J/cm^2^ and up to 250 mW/cm^2^ at the level of the scalp (Huang et al., 2011; Hamblin, 2016; Yang et al., 2021; Dole et al., 2023; Mitrofanis et al., 2024). Pulsed stimulation is also thought to result in better effects, both by producing lesser heating of the tissues and by penetrating further through the skin barrier (Selestin Raja et al., 2024).

One issue that appears to be more consistent across the literature is that there is a clear biphasic dose-response relationship. That is, medium doses (in the middle of the bell-curve) are more effective than either lower or higher doses (at either end of the curve). In fact, several studies have indicated that with higher doses, there could be an inhibitory, but not toxic, effect of light on the cells (Huang et al., 2011; Hamblin, 2016; Yang et al., 2021). As with many other types of therapies, from electrical to pharmaceutical, finding that “sweet-spot” of light treatment in the middle of the bell curve is the key to positive outcomes.

### Does light treatment influence the aging process?

There have been many preclinical studies reporting beneficial outcomes of light treatment on animal models of aging, almost as many as those examining animal models of the neurodegenerative diseases, Alzheimer’s and Parkinson’s diseases (Hamblin, 2016; Mitrofanis, 2019; see Rodríguez-Fernández et al., 2024 for a recent review). The most common of the aging models involves mice or rats that have been aged between 12 and 24 months. These studies, as with those on animal models of neurodegenerative diseases, have reported the following beneficial changes:

- ↓ Inflammation (El Massri et al., 2018; Cardoso et al., 2022; Hosseini et al., 2022);

- ↓ Cell death proteins (Salehpour et al., 2017; Cardoso et al., 2021a);

- ↑ Mitochondrial function (Kokkinopoulos et al., 2013; Salehpour et al., 2017; Sivapathasuntharam et al., 2017; Weinrich et al., 2018);

- ↑ Glucose metabolism (Cardoso et al., 2021a);

- ↑ Spatial and episodic-like memory (Hosseini et al., 2022; Michalikova et al., 2008; Salehpour et al., 2017; Cardoso et al., 2021b).

There have also been several explorations into the effect of transcranial light in elderly humans (Cardoso et al., 2021c; Gao et al., 2023). In general, these studies have shown many beneficial outcomes, including:

- ↑ Cerebral blood flow (Vargas et al., 2017), oxidized cytochrome-c-oxidase and [HbO]/[Hb] ratio (Saucedo et al., 2021) at rest;

- ↑ Plasma brain–derived growth factor levels (de Oliveira et al., 2024);

- ↑ Resting-state electroencephalogram α, β, γ power (Zomorrodi et al., 2019);

- ↓ Task-related prefrontal hemodynamic response with near infrared spectroscopy (Lee and Chan, 2023);

- ↑ Functional connectivity, local and global efficiency (Yang et al., 2025);

- ↑ Cognition: general improvement in scores using neuropsychological tests such as the Mini-Mental State Examination or the Montreal Cognitive Assessment tests (Nizamutdinov et al., 2021; de Oliveira et al., 2024), an increase in inhibition and flexibility (Chan et al., 2019), together with improvements in working memory (Qu et al., 2022; Lee and Chan, 2023; Yang et al., 2025).

The parameters used by these clinical studies have been varied (see above), including wavelengths from 633–660 nm to 850–870 nm to 1064 nm, power intensities on the scalp from 10 to 250 mW/cm^2^, and dosages from 20 to 120 J/cm^2^. All the studies have been rather short-term, with treatments extending from a single session to three sessions per week for four to eight weeks only (Vargas et al., 2017; Chan et al., 2019; Saucedo et al., 2021; Qu et al., 2022; Lee and Chan, 2023; de Oliveira et al., 2024).

In terms of overall function, light has been suggested to activate mechanisms that help focus attention and/or to help restore the overall balance of function and connectivity across any given system, particularly if it becomes dysfunctional, as in aging. There have also been many studies indicating light-induced benefits in various neurological diseases, including depression, Alzheimer’s and Parkinson’s diseases, stroke, and traumatic brain injury; in each of these conditions, light treatment has been shown to have positive outcomes (Naeser and Hamblin, 2015; Hamblin, 2016; Naeser et al., 2023; Mitrofanis and Henderson, 2020; Dole et al., 2023).

## Conclusions and Hypothesis

In animal models, nearly all the features that characterize the cellular changes in the brain associated with aging (**Figures 1** and **3**) have been shown to be improved with light treatment (**Figures 5** and **6**). There are also some very encouraging indications that light treatment can be effective in humans, improving cerebral blood flow, cognition, various metabolic parameters, and functional activity in aged subjects. However, to date clinical studies have been rather short-term (from one single session to several weeks) and using only minimal doses of transcranial light. What is need at this stage is for placebo-controlled clinical studies to trace functional changes over a longer time-frame (i.e., months to years) and for a more consistent and extended application of light (i.e., daily). The results from such long-term, more rigorous clinical studies will provide clearer insights as to whether light treatment can be effective in improving brain function and cognition in aged individuals. They will make great strides into the development of this non-invasive, non-pharmacological, easy to use and totally safe treatment to improve the quality of life of this every-growing sector of our community.

Taking it a step further, we would like to offer a global hypothesis for the effect of transcranial light on the aging process. We suggest that light treatment will increase the resilience of neurons against the aging process, improving their survival and function, together with reducing the patterns of inflammation. Further, these light-induced cellular improvements result in better overall brain function. Light treatment could potentially act on neurovascular coupling, helping to improve the patterns of functional connectivity between and within different large-scale resting state networks, together with the patterns of activation after undertaking a particular task, whether it be movement of cognition. In essence, we propose that light will help facilitate a reduction in the functional impairments and compensations evident in the aged brain. We suggest that light can promote a restoration of patterns of activation and functional connectivity in the aged brain (**Figure 4**) and help prevent the onset of neurological disorders such as stroke and neurodegenerative disease.

Finally, we should mention the potential application of near infrared light in the field of tissue engineering. Given its potential to drive anti-inflammatory and anti-oxidant mechanisms, to increase growth factors and stimulate cell proliferation, it has been proven useful in various applications of tissue regeneration in different tissue types (see Selestin-Raja et al., 2024 for a recent review). Whereas the results on the neural tissues are rather scarce, it has been used in a rat model of spinal cord injury (Janzadeh et al., 2017). Whether it could be used for other applications in the neural system will be certainly subject to further investigations.

**Additional file:** *Open peer review report 1.*

OPEN PEER REVIEW REPORT 1

## Data Availability

*All relevant data are within the paper and its Additional files.*

## References

[R1] Abdo A, Ersen A, Sahin M (2013). Near-infrared light penetration profile in the rodent brain. J Biomed Opt.

[R2] Achard S, Bullmore E (2007). Efficiency and cost of economical brain functional networks. PLoS Comput Biol.

[R3] Aron L, Zullo J, Yankner BA (2022). The adaptive aging brain. Curr Opin Neurobiol.

[R4] Anatürk M, Kaufmann T, Cole JH, Suri S, Griffanti L, Zsoldos E, Filippini N, Singh-Manoux A, Kivimäki M, Westlye LT, Ebmeier KP, de Lange AG (2020). Prediction of brain age and cognitive age: quantifying brain and cognitive maintenance in aging. Human Brain Mapping.

[R5] Atkins JL, Jylhävä J, Pedersen NL, Magnusson PK, Lu Y, Wang Y, Hägg S, Melzer D, Williams DM, Pilling LC (2021). A genome-wide association study of the frailty index highlights brain pathways in ageing. Aging Cell.

[R6] Azam S, Haque ME, Balakrishnan R, Kim IS, Choi DK (2021). The ageing brain: molecular and cellular basis of neurodegeneration. Front Cell Dev Biol.

[R7] Beck D, de Lange AG, Pedersen ML, Alnaes D, Maximov II, Voldsbekk I, Richard G, Sanders AM, Ulrichsen KM, Dørum ES, Kolskår KK, Høgestøl EA, Steen NE, Djurovic S, Andreassen OA, Nordvik JE, Kaufmann T, Westlye LT (2021). Cardiometabolic factors associated with brain age and accelerate brain ageing. Hum Brain Mapp.

[R8] Bibb SA, Y EJ, Molloy MF, LaRocco J, Resnick P, Reeves K, Luan Phan K, Krishna S, Saygin ZM (2025). Pilot study comparing effects of infrared neuromodulation and transcranial magnetic stimulation using magnetic resonance imaging. Front Hum Neurosci.

[R9] Bicknell B, Liebert A, McLachlan CS, Kiat H (2022). Microbiome changes in humans with Parkinson’s disease after photobiomodulation therapy: a retrospective study. J Pers Med.

[R10] Blivet G, Meunier J, Roman FJ, Touchon J (2018). Neuroprotective effect of a new photobiomodulation technique against Aβ25–35 peptide–induced toxicity in mice: novel hypothesis for therapeutic approach of Alzheimer’s disease suggested. Alzheimers Dement.

[R11] Boyer D, Hu A, Warrow D, Xavier S, Gonzalez V, Lad E, Rosen RB, Do D, Schneiderman T, Hu A, Munk MR, Jaffe G, Tedford SE, Croissant CL, Walker M, Rückert R, Tedford CE (2023). LIGHTSITE III: 13-month efficacy and safety evaluation of multiwavelength photobiomodulation in nonexudative (dry) age-related macular degeneration using the LumiThera valeda light delivery system. Retina.

[R12] Byrnes KR, Waynant RW, Ilev IK, Wu X, Barna L, Smith K, Heckert R, Gerst H, Anders JJ (2005). Light promotes regeneration and functional recovery and alters the immune response after spinal cord injury. Lasers Surg Med.

[R13] Cabeza R (2002). Hemispheric asymmetry reduction in older adults: the HAROLD model. Psychol Aging.

[R14] Cardoso FDS, Gonzalez-Lima F, Gomes da Silva S (2021). Photobiomodulation for the aging brain. Ageing Res Rev.

[R15] Cardoso FDS, Mansur FCB, Lopes-Martins RÁB, Gonzalez-Lima F, Gomes da Silva S (2021). Transcranial laser photobiomodulation improves intracellular signaling linked to cell survival, memory and glucose metabolism in the aged brain: a preliminary study. Front Cell Neurosci.

[R16] Cardoso FDS, de Souza Oliveira Tavares C, Araujo BHS, Mansu F, Lopes-Martins RÁB, Gomes da Silva S (2021). Improved spatial memory and neuroinflammatory profile changes in aged rats submitted to photobiomodulation therapy. Cell Mol Neurobiol.

[R17] Cardoso FDS, Salehpour F, Coimbra NC, Gonzalez-Lima F, Gomes Da Silva S (2022). Photobiomodulation for the treatment of neuroinflammation: a systematic review of controlled laboratory animal studies. Front Neurosci.

[R18] Cassano P, Caldieraro MA, Norton R, Mischoulon D, Trinh NH, Nyer M, Dording C, Hamblin MR, Campbell B, Iosifescu DV (2019). Reported side effects, weight and blood pressure, after repeated sessions of transcranial photobiomodulation. Photobiomodul Photomed Laser Surg.

[R19] Chan AS, Lee TL, Yeung MK, Hamblin MR (2019). Photobiomodulation improves the frontal cognitive function of older adults. Int J Geriat Psychiatry.

[R20] Chan AS, Lee TL, Hamblin MR, Cheung MC (2021). Photobiomodulation enhances memory processing in older adults with mild cognitive impairment: a functional near-infrared spectroscopy study. J Alzheimers Dis.

[R21] Coelho DRA, Vieira WF, Hurtado Puerto AM, Gersten MB, Collins KA, Peterson A, Siu K, Tural U, Iosifescu DV, Cassano P (2025). Dose-dependent tolerability and safety of transcranial photobiomodulation: a randomized controlled trial. Lasers Med Sci.

[R22] Colombo E, Signore A, Aicardi S, Zekiy A, Utyuzh A, Benedicenti S, Amaroli A (2021). Experimental and clinical applications of red and near-infrared photobiomodulation on endothelial dysfunction: a review. Biomedicines.

[R23] Damoiseaux J (2017). Effects of aging on functional and structural brain connectivity. NeuroImage.

[R24] Darlot F, Moro C, El Massri N, Chabrol C, Johnstone DM, Reinhart F, Agay D, Torres N, Bekha D, Auboiroux V, Costecalde T, Peoples CL, Anastascio HDT, Shaw VE, Stone J, Mitrofanis J, Benabid AL (2016). Near-infrared light is neuroprotective in a monkey model of Parkinson disease. Ann Neurol.

[R25] Davis SW, Dennis NA, Daselaar SM, Fleck MS, Cabeza R (2008). Que PASA? The posterior-anterior shift in aging. Cereb Cortex.

[R26] de Oliveira B, Lins E, Kunde N, Salgado ASI, Martins L, Bobinski F, Viera W, Cassano P, Quialheiro A, Martins D (2024). Transcranial photobiomodulation increases cognition and serum BDNF levels in adults over 50 years: a randomized, double-blind, placebo- controlled trial. J Photochem Photobiol B.

[R27] Deery HA, Di Paolo R, Moran C, Egan GF, Jamadar SD (2023). The older adult brain is less modular, more integrated, and less efficient at rest: a systematic review of large-scale resting-state functional brain networks in aging. Psychophysiology.

[R28] Deery HA, Di Paolo R, Moran C, Egan GF, Jamadar SD (2023). Lower brain glucose metabolism in normal ageing is predominantly frontal and temporal: a systematic review and pooled effect size and activation likelihood estimates meta-analyses. Hum Brain Mapp.

[R29] Denburg NL, Hedgcock WM, Hess TM, Strough J, Löckenhoff CE (2015). Age-associated executive dysfunction, the prefrontal cortex, and complex decision making. Aging and decision making.

[R30] DeTaboada L, Ilic S, Leichliter-Martha S, Oron U, Oron A, Streeter J (2006). Transcranial application of low-energy laser irradiation improves neurological deficits in rats following acute stroke. Lasers Surg Med.

[R31] Dmochowski GM, Shereen AD, Berisha D, Dmochowski JP (2020). Near-infrared light increases functional connectivity with a non-thermal mechanism. Cereb Cortex Commun.

[R32] Dole M, Auboiroux V, Langar L, Mitrofanis J (2023). A systematic review of the effects of transcranial photobiomodulation on brain activity in humans. Rev Neurosci.

[R33] Dole M, Bleuet P, Auboiroux V, Billères M, Mitrofanis J (2024). Monte Carlo simulations of a multisource transcranial photobiomodulation helmet device: application to young and aged brains. Adv Technol Neurosci.

[R34] Dos Santos Cardoso F, Mansur FCB, Silva Araujo BH, Gonzalez-Lima F, Gomes da Silva S (2022). Photobiomodulation improves the infammatory response and intracellular signaling proteins linked to vascular function and cell survival in the brain of aged rats. Mol Neurobiol.

[R35] El Khoury H, Mitrofanis J, Henderson LA (2019). Exploring the effects of near infrared light on resting and evoked brain activity in humans using magnetic resonance imaging. Neuroscience.

[R36] El Massri N, Weinrich TW, Hoh Kam J, Jeffery G, Mitrofanis J (2018). Photobiomodulation reduces gliosis in the basal ganglia of aged mice. Neurobiol Aging.

[R37] Fabiani M, Gordon BA, Maclin EL, Pearson MA, Brumback-Peltz CR, Low KA, McAuley E, Sutton BP, Kramer AF, Gratton G (2014). Neurovascular coupling in normal aging: a combined optical, ERP and fMRI study. Neuroimage.

[R38] Gao Y, An R, Huang X, Liu W, Yang C, Wan Q (2023). Effectiveness of photobiomodulation for people with age-related cognitive impairment: a systematic review and meta-analysis. Lasers Med Sci.

[R39] Gonzales MM, Garbarino VR, Kautz TF, Song X, Lopez-Cruzan M, Linehan L, Van Skike CE, De Erausquin GA, Galvan V, Orr ME, Musi N, He Y, Bateman RJ, Wang CP, Seshadri S, Kraig E, Kellogg D (2025). Rapamycin treatment for Alzheimer’s disease and related dementias: a pilot phase 1 clinical trial. Commun Med (Lond).

[R40] Gonzalez-Lima F, Barrett DW (2014). Augmentation of cognitive brain functions with transcranial lasers. Front Syst Neurosci.

[R41] Grady C (2012). The cognitive neuroscience of ageing. Nat Rev Neurosci.

[R42] Grover F, Weston J, Weston M (2017). Acute effects of near infrared light therapy on brain state in healthy subjects as quantified by qEEG measures. Photomed Laser Surg.

[R43] Haeussinger FB, Heinzel S, Hahn T, Schecklmann M, Ehlis AC, Fallgatter AJ (2011). Simulation of near-infrared light absorption considering individual head and prefrontal cortex anatomy: implications for optical neuroimaging. PLoS One.

[R44] Hamblin MR (2016). Shining light on the head: photobiomodulation for brain disorders. BBA Clin.

[R45] Hamblin MR (2017). Mechanisms and applications of the anti-inflammatory effects of photobiomodulation. AIMS Biophys.

[R46] Hamblin MR (2018). Photobiomodulation for traumatic brain injury and stroke. J Neurosci Res.

[R47] Hamblin MR, Liebert A (2022). Photobiomodulation therapy mechanisms beyond cytochrome c oxidase. Photobiomodul Photomed Laser Surg.

[R48] He L, Wang X, Zhuang K, Qiu J (2020). Decreased dynamic segregation but increased dynamic integration of the resting-state functional networks during normal aging. Neuroscience.

[R49] Henderson TA, Morries LD (2015). Near-infrared photonic energy penetration: can infrared phototherapy effectively reach the human brain?. Neuropsychiatr Dis Treat.

[R50] Hennessy M, Hamblin MR (2017). Photobiomodulation and the brain: a new paradigm. J Opt.

[R51] Hosseini L, Farazi N, Erfani M, Mahmoudi J, Akbari M, Hosseini SH, Sadigh-Eteghad S (2022). Effect of transcranial near-infrared photobiomodulation on cognitive outcomes in D-galactose/AlCl3 induced brain aging in BALB/c mice. Lasers Med Sci.

[R52] Hu Z, Qu X, Li L, Zhou X, Yang Q, Dong Q, Liu H, Li X, Han Y, Niu H (2023). Repeated photobiomodulation induced reduction of bilateral cortical hemodynamic activation during a working memory task in healthy older adults. IEEE J Biomed Health Inform.

[R53] Huang YY, Sharma SK, Carroll J, Hamblin MR (2011). Biphasic dose response in low level light therapy - an update. Dose Response.

[R54] Ilic S, Leichliter S, Streeter J, Oron A, DeTaboada L, Oron U (2006). Effects of power densities, continuous and pulse frequencies, and number of sessions of low-level laser therapy on intact rat brain. Photomed Laser Surg.

[R55] Jagdeo JR, Adams LE, Brody NI, Siegel DM (2012). Transcranial red and near infrared light transmission in a cadaveric model. PLoS One.

[R56] Jahan A, Nazari MA, Mahmoudi J, Salehpour F, Salimi MM (2019). Transcranial near-infrared photobiomodulation could modulate brain electrophysiological features and attentional performance in healthy young adults. Lasers Med Sci.

[R57] Janzadeh A, Sarveazad A, Yousefifard M, Dameni S, Sahraneshin Samani F, Mokhtarian K, Nasirinezhad F (2017). Combine effect of chondroinitase ABC and low-level laser (660nm) on spinal cord injury model in adult male rats. Neuropeptides.

[R58] Jin M, Cai SQ (2023). Mechanisms underlying brain aging under normal and pathological conditions. Neurosci Bull.

[R59] Johnstone DM, El Massri N, Moro C, Spana S, Wang XS, Torres N, Chabrol C, De Jaeger X, Reinhart F, Purushothuman S, Benabid A-L, Stone J, Mitrofanis J (2014). Indirect application of near infrared light induces neuroprotection in a mouse model of parkinsonism - an abscopal neuroprotective effect. Neuroscience.

[R60] Kokkinopoulos I, Colman A, Hogg C, Heckenlively J, Jeffery G (2013). Age-related retinal inflammation is reduced by 670 nm light via increased mitochondrial membrane potential. Neurobiol Aging.

[R61] Konstantinović LM, Jelić MB, Jeremić A, Stevanović VB, Milanović SD, Filipović SR (2013). Transcranial application of near-infrared low-level laser can modulate cortical excitability. Lasers Surg Med.

[R62] Kraig E, Linehan LA, Liang H, Romo TQ, Liu Q, Wu Q, Benavides AD, Curiel TJ, Javors MA, Musi N, Chiodo L, Koek W, Gelfond JAL, Kellog DL (2018). A randomized control trial to establish the feasibility and safety of Rapamycin treatment in an older human cohort: immunological, physical performance and cognitive effects. Exp Gerontol.

[R63] Lapchak PA, Boitano PD, Butte PV, Fisher DJ, Hölscher T, Ley EJ, Nuño M, Voie AH, Rajput PS (2015). Transcranial near-infrared laser transmission (NILT) profiles (800 nm): systematic comparison in four common research species. PLoS One.

[R64] Lavanga M, Stumme J, Yalcinkaya BH, Fousek J, Jockwitz C, Sheheitli H, Bittner N, Hashemi M, Petkoski S, Caspers S, Jirsa V (2023). The virtual aging brain: causal inference supports interhemispheric dedifferentiation in healthy aging. Neuroimage.

[R65] Lee DJW, Hodzic Kuerec A, Maier AB (2024). Targeting ageing with rapamycin and its derivatives in humans: a systematic review. Lancet Healthy Longev.

[R66] Lee TL, Chan AS (2023). Photobiomodulation may enhance cognitive efficiency in older adults: a functional near-infrared spectroscopy study. Front Aging Neurosci.

[R67] Li D, Liu S, Yu T, Liu Z, Sun S, Bragin D, Shirokov A, Navolokin N, Bragina O, Hu Z, Kurths J, Fedosov I, Blokhina I, Dubrovski A, Khorovodov A, Terskov A, Tzoy M, Semyachkina-Glushkovskaya O, Zhu D (2023). Photostimulation of brain lymphatics in male newborn and adult rodents for therapy of intraventricular hemorrhage. Nat Commun.

[R68] Li Z, Zhang Z, Ren Y, Wang Y, Fang J, Yue H, Ma S, Guan F (2021). Aging and age-related diseases: from mechanisms to therapeutic strategies. Biogerontology.

[R69] Li Z, Zhao Y, Hu Y, Li Y, Zhang K, Gao Z, Tan L, Jia H, Cong J, Liu H, Li X, Cao A, Cui Z, Zhao C (2024). Transcranial low-level laser stimulation in the near-infrared-II region (1064 nm) for brain safety in healthy humans. Brain Stimul.

[R70] Liang Z, Lei T, Wang S, Li P, Chen B, Pan D, Zhang Y, Zuo X, Wang X, Luo Z, Hu X, Ding T, Wang Z (2022). Clinical safety study of photobiomodulation in acute spinal cord injury by scattering fiber. Lasers Med Sci.

[R71] Liu C, Jing J, Jiang J, Wen W, Zhu W, Li Z, Pan Y, Cai X, Liu H, Zhou Y, Meng X, Zhang J, Wang Y, Li H, Jiang Y, Zheng H, Wang S, Niu H, Kochan N, Brodaty H, Wei T, Sachdev P, Liu T, Wang Y (2024). Relationships between brain structure-function coupling in normal aging and cognition: a cross-ethnicity population-based study. Neuroimage.

[R72] López-Otín C, Blasco MA, Partridge L, Serrano M, Kroemer G (2013). The hallmarks of aging. Cell.

[R73] Mannick JB, Morris M, Hockey HU, Roma G, Beibel M, Kulmatycki K, Watkins M, Shavlakadze T, Zhou W, Quinn D, Glass DJ, Klickstein LB (2018). TORC1 inhibition enhances immune function and reduces infections in the elderly. Sci Transl Med.

[R74] Marseglia A, Dartora C, Samuelsson J, Poulakis K, Mohanty R, Shams S, Lindberg O, Rydén L, Sterner TR, Skoog J, Zettergren A, Kern S, Skoog I, Westman E (2024). Biological brain age and resilience in cognitively unimpaired 70-year-old individuals. Alzheimers Dement.

[R75] Martins R, Joanette Y, Monchi O (2015). The implications of age-related neurofunctional compensatory mechanisms in executive function and language processing including the new temporal hypothesis for compensation. Front Hum Neurosci.

[R76] Mattay VS, Fera F, Tessitore A, Hariri AR, Das S, Callicott JH, Weinberger DR (2002). Neurophysiological correlates of age-related changes in human motor function. Neurology.

[R77] McCarthy TJ, De Taboada L, Hildebrandt PK, Ziemer EL, Richieri SP, Streeter J (2010). Long-term safety of single and multiple infrared transcranial laser treatments in Sprague-Dawley rats. Photomed Laser Surg.

[R78] Michalikova S, Ennaceur A, van Rensburg R, Chazot PL (2008). Emotional responses and memory performance of middle-aged CD1 mice in a 3D maze: Effects of low infrared light. Neurobiol Learn Mem.

[R79] Mitrofanis J (2019). Run in the light: exploring exercise and photobiomodulation in Parkinson’s disease.

[R80] Mitrofanis J, Jeffery G (2018). Does photobiomodulation influence ageing?. Aging (Albany NY).

[R81] Mitrofanis J, Henderson LA (2020). How and why does photobiomodulation change brain activity?. Neural Regen Res.

[R82] Mitrofanis J, Stone J, Hamblin MR, Magistretti P, Benabid AL, Jeffery G (2024). A spotlight on dosage and subject selection for effective neuroprotection: exploring the central role of mitochondria. Neural Regen Res.

[R83] Moro C, El Massri N, Torres N, Ratel D, De Jaeger X, Chabrol C, Perraut F, Bourgerette A, Berger M, Purushothuman S, Johnstone DM, Stone J, Mitrofanis J, Benabid AL (2014). Photobiomodulation inside the brain: a novel method of applying near-infrared light intracranially and its impact on dopaminergic cell survival in MPTP-treated mice. J Neurosurg.

[R84] Moro C, El Massri N, Darlot F, Torres N, Chabrol C, Agay D, Auboiroux V, Johnstone DM, Stone J, Mitrofanis J, Benabid AL (2016). Effects of a higher dose of near-infrared light on clinical signs and neuroprotection in a monkey model of Parkinson’s disease. Brain Res.

[R85] Na D, Zhang Z, Meng M, Li M, Gao J, Kong J, Zhang G, Guo Y (2025). Energy metabolism and brain aging: strategies to delay neuronal degeneration. Cell Mol Neurobiol.

[R86] Naeser MA, Hamblin MR (2015). Traumatic brain injury: a major medical problem that could be treated using transcranial, red/near-infrared LED photobiomodulation. Photomed Laser Surg.

[R87] Naeser MA, Martin PI, Ho MD, Krengel MH, Bogdanova Y, Knight JA, Hamblin MR, Fedoruk A, Poole LG, Cheng C, Koo B (2023). Transcranial photobiomodulation treatment: significant improvements in four ex-football players with possible chronic traumatic encephalopathy. J Alzheimers Dis Rep.

[R88] Nizamutdinov D, Qi X, Berman MH, Dougal G, Dayawansa S, Wu E, Yi SS, Stevens AB, Huang JH (2021). Transcranial near infrared light stimulations improve cognition in patients with dementia. Aging Dis.

[R89] O’Shea A, Cohen RA, Porges EC, Nissim NR, Woods AJ (2016). Cognitive aging and the hippocampus in older adults. Front Aging Neurosci.

[R90] Park DC, Lautenschlager G, Hedden T, Davidson NS, Smith AD, Smith PK (2002). Models of visuospatial and verbal memory across the adult life span. Psychol Aging.

[R91] Park DC, Reuter-Lorenz P (2009). The adaptive brain: aging and neurocognitive scaffolding. Annu Rev Psychol.

[R92] Parvin P, Hamid Reza D, Atefeh B, Davoudi P (2023). Tissue thermal response in photobiomodulation therapy: a theoretical study. SciBase Dent Oral Sci.

[R93] Peters R (2006). Ageing and the brain. Postgrad Med J.

[R94] Pettigrew C, Soldan A (2019). Defining cognitive reserve and implications for cognitive aging. Curr Neurol Neurosci Rep.

[R95] Pitzschke A, Lovisa B, Seydoux O, Zellweger M, Pfleiderer M, Tardy Y, Wagnières G (2015). Red and NIR light dosimetry in the human deep brain. Phys Med Biol.

[R96] Qu X, Li L, Zhou X, Dong Q, Liu Hanli, Liu Hesheng, Yang Q, Han Y, Niu H (2022). Repeated transcranial photobiomodulation improves working memory of healthy older adults: behavioral outcomes of poststimulation including a three-week follow-up. Neurophotonics.

[R97] Quirk BJ, Whelan HT (2011). Near-infrared irradiation photobiomodulation: the need for basic science. Photomed Laser Surg.

[R98] Reinhart F, El Massri N, Chabrol C, Cretallaz C, Johnstone DM, Torres N, Darlot F, Costecalde T, Stone J, Mitrofanis J, Benabid AL, Moro C (2016). Intracranial application of near-infrared light in a hemi-parkinsonian rat model: the impact on behavior and cell survival. J Neurosurg.

[R99] Rodríguez-Fernández L, Zorzo C, Arias JL (2024). Photobiomodulation in the aging brain: a systematic review from animal models to humans. GeroScience.

[R100] Salehpour F, Ahmadian N, Rasta SH, Farhoudi M, Karimi P, Sadigh-Eteghad S (2017). Transcranial low-level laser therapy improves brain mitochondrial function and cognitive impairment in D-galactose-induced aging mice. Neurobiol Aging.

[R101] Salthouse TA (1996). The processing-speed theory of adult age differences in cognition. Psychol Rev.

[R102] Saltmarche AE, Naeser MA, Ho KF, Hamblin MR, Lim L (2017). Significant improvement in cognition in mild to moderately severe dementia cases treated with transcranial plus intranasal photobiomodulation: case series report. Photomed Laser Surg.

[R103] San Miguel M, Martin KL, Stone J, Johnstone DM (2019). Photobiomodulation mitigates cerebrovascular leakage induced by the parkinsonian neurotoxin MPTP. Biomolecules.

[R104] Saucedo CL, Courtois EC, Wade ZS, Kelley MN, Kheradbin N, Barrett DW, Gonzalez-Lima F (2021). Transcranial laser stimulation: mitochondrial and cerebrovascular effects in younger and older healthy adults. Brain Stimul.

[R105] Selestin Raja I, Kim C, Oh N, Park JH, Won Hong S, Sun Kang M, Mao C, Han DW (2024). Tailoring photobiomodulation to enhance tissue regeneration. Biomaterials.

[R106] Semyachkina-Glushkovskaya O, Penzel T, Blokhina I, Khorovodov A, Fedosov I, Yu T, Karandin G, Evsukova A, Elovenko D, Adushkina V, Shirokov A, Dubrovskii A, Terskov A, Navolokin N, Tzoy M, Ageev V, Agranovich I, Telnova V, Tsven A, Kurths J (2021). Night photostimulation of clearance beta-amyloid from mouse brain: new strategies in preventing Alzheimer’s disease. Cells.

[R107] Shahdadian S, Wang X, Liu H (2024). Directed physiological networks in the human prefrontal cortex at rest and post transcranial photobiomodulation. Sci Rep.

[R108] Shamloo S, Defensor E, Ciari P, Ogawa G, Vidano L, Lin JS, Fortkort JA, Shamloo M, Barron AE (2023). The anti-inflammatory effects of photobiomodulation are mediated by cytokines: evidence from a mouse model of inflammation. Front Neurosci.

[R109] Shan YC, Fang W, Chang YC, Chang WD, Wu JH (2021). Effect of near-infrared pulsed light on the human brain using electroencephalography. Evid Based Complement Alternat Med.

[R110] Shaw VE, Spana S, Ashkan K, Benabid AL, Stone J, Baker GE, Mitrofanis J (2010). Neuroprotection of midbrain dopaminergic cells in MPTP-treated mice after near-infrared light treatment. J Comp Neurol.

[R111] Sivapathasuntharam C, Sivaprasad S, Hogg C, Jeffery G (2017). Aging retinal function is improved by near infrared light (670 nm) that is associated with corrected mitochondrial decline. Neurobiol Aging.

[R112] Stern Y (2012). Cognitive reserve in ageing and Alzheimer’s disease. Lancet Neurol.

[R113] Stern Y, Albert M, Barnes CA, Cabeza R, Pascual-Leone A, Rapp PR (2023). A framework for concepts of reserve and resilience in aging. Neurobiol Aging.

[R114] Stone J, Johnstone DM, Mitrofanis J, O’Rourke M (2015). The mechanical cause of age-related dementia (Alzheimer’s disease): the brain is destroyed by the pulse. J Alzheimers Dis.

[R115] Stone J, Mitrofanis J, Johnstone DM, Falsini B, Bisti S, Adam P, Nuevo AB, George-Weinstein M, Mason R, Eells J (2019). Acquired resilience: an evolved system of tissue protection in mammals. Dose Response.

[R116] Tata DB, Waynant RW (2012). Laser therapy: a review of its mechanism of action and potential medical applications. Laser Photonics Rev.

[R117] Tamatta R, Pai V, Jaiswal C, Singh I, Singh AK (2025). Neuroinflammaging and the immune landscape: the role of autophagy and senescence in aging brain. Biogerontology.

[R118] Tedford CE, DeLapp S, Jacques S, Anders J (2015). Quantitative analysis of transcranial and intraparenchymal light penetration in human cadaver brain tissue. Lasers Surg Med.

[R119] Tromp D, Dufour A, Lithfous S, Pebayle T, Després O (2015). Episodic memory in normal aging and Alzheimer disease: insights from imaging and behavioral studies. Ageing Res Rev.

[R120] Truong NCD, Wang X, Wanniarachchi H, Liu H (2022). Enhancement of frequency-specific hemodynamic power and functional connectivity by transcranial photobiomodulation in healthy humans. Front Neurosci.

[R121] Urquhart EL, Wanniarachchi H, Wang X, Gonzalez-Lima F, Alexandrakis G, Liu H (2020). Transcranial photobiomodulation-induced changes in human brain functional connectivity and network metrics mapped by whole-head functional near-infrared spectroscopy in vivo. Biomed Opt Express.

[R122] Van Ruitenbeek P, Santos Monteiro T, Chalavi S, King BR, Cuypers K, Sunaert S, Peeters R, Swinnen SP (2023). Interactions between the aging brain and motor task complexity across the lifespan: balancing brain activity resource demand and supply. Cerebral Cortex.

[R123] Varela-López B, Cruz-Gomez ÁJ, Lojo-Seoane C, Díaz F, Pereiro AX, Zurrón M, Lindín M, Galdo-Álvarez S (2022). Cognitive reserve, neurocognitive performance, and high-order resting-state networks in cognitively unimpaired aging. Neurobiol Aging.

[R124] Vargas E, Barrett DW, Saucedo CL, Huang LD, Abraham JA, Tanaka H, Haley AP, Gonzalez-Lima F (2017). Beneficial neurocognitive effects of transcranial laser in older adults. Lasers Med Sci.

[R125] Wang L, Zhang Y, Zhang J, Sang L, Li P, Yan R, Qiu M, Liu C (2019). Aging changes effective connectivity of motor networks during motor execution and motor imagery. Front Aging Neurosci.

[R126] Wang X, Tian F, Reddy DD, Nalawade SS, Barrett DW, Gonzalez-Lima F, Liu H (2017). Up-regulation of cerebral cytochrome-c-oxidase and hemodynamics by transcranial infrared laser stimulation: a broadband near-infrared spectroscopy study. J Cereb Blood Flow Metab.

[R127] Wang X, Wanniarachchi H, Wu A, Gonzalez-Lima F, Liu H (2021). Transcranial photobiomodulation and thermal stimulation induce distinct topographies of EEG alpha and beta power changes in healthy humans. Sci Rep.

[R128] Weinrich T, Hogg C, Jeffery G (2018). The temporal sequence of improved mitochondrial function on the dynamics of respiration, mobility and cognition in aged Drosophila. Neurobiol Aging.

[R129] Wen X, He H, Dong L, Chen J, Yang J, Guo H, Luo C, Yao D (2020). Alterations of local functional connectivity in lifespan: a resting-state fMRI study. Brain Behav.

[R130] Wiseman SJ, Booth T, Ritchie SJ, Cox SR, Muñoz Maniega S, Valdés Hernández MDC, Dickie DA, Royle NA, Starr JM, Deary IJ, Wardlaw JM, Bastin ME (2018). Cognitive abilities, brain white matter hyperintensity volume, and structural network connectivity in older age. Hum Brain Mapp.

[R131] Wittens MMJ (2024). Brain age as a biomarker for pathological versus healthy ageing – a REMEMBER study. Alzheimers Res Ther.

[R132] Wrigglesworth J, Yaacob N, Ward P, Woods RL, McNeil J, Storey E, Egan G, Murray A, Shah RC, Jamadar SD, Trevaks R, Ward S, Harding IH, Ryan J, ASPREE investigator group (2022). Brain-predicted age difference is associated with cognitive processing in later-life. Neurobiol Aging.

[R133] Xie K, El Khoury H, Mitrofanis J, Austin PJ (2022). A systematic review of the effect of photobiomodulation on the neuroinflammatory response in animal models of neurodegenerative diseases. Rev Neurosci.

[R134] Xuan W, Agrawal T, Huang L, Gupta GK, Hamblin MR (2015). Low-level laser therapy for traumatic brain injury in mice increases brain derived neurotrophic factor (BDNF) and synaptogenesis. J Biophoton.

[R135] Yang M, Yang Z, Wang P, Sun Z (2021). Current application and future directions of photobiomodulation in central nervous diseases. Neural Regen Res.

[R136] Yang Q, Qu X, Sheng C, Zhao X, Chen G, Wang X, Li Y, Du W, Wang X, Sun Y, Li X, Niu H, Han Y (2025). Transcranial photobiomodulation improves functional brain networks and working memory in healthy older adults: an fNIRS study. Neuroimage.

[R137] Yi F, Yuan J, Somekh J, Peleg M, Zhu YC, Jia Z, Wu F, Huang Z (2025). Genetically supported targets and drug repurposing for brain aging: a systematic study in the UK Biobank. Sci Adv.

[R138] Yuan Y, Cassano P, Pias M, Fang Q (2020). Transcranial photobiomodulation with near-infrared light from childhood to elderliness: simulation of dosimetry. Neurophotonics.

[R139] Zanto TP, Gazzaley A (2019). Aging of the frontal lobe. Handb Clin Neurol.

[R140] Zhang H, Cao P, Mak HFK, Hui ES (2024). The structural-functional-connectivity coupling of the aging brain. GeroScience.

[R141] Zheng W, Li K, Zhong M, Wu K, Zhou L, Huang J, Liu L, Chen Z (2024). Mitophagy activation by rapamycin enhances mitochondrial function and cognition in 5xFAD mice. Behav Brain Res.

[R142] Zomorrodi R, Loheswaran G, Pushparaj A, Lim L (2019). Pulsed near infrared transcranial and intranasal photobiomodulation significantly modulates neural oscillations: a pilot exploratory study. Sci Rep.

[R143] Zonneveld HI, Pruim RH, Bos D, Vrooman HA, Muetzel RL, Hofman A, Rombouts SA, van der Lugt A, Niessen WJ, Ikram MA, Vernooij MW (2019). Patterns of functional connectivity in an aging population: The Rotterdam Study. Neuroimage.

