# Supplementary material for: Lights for aging: Can photobiomodulation restore functionality in the cerebral networks of aged individuals?
Source: Neural Regen Res. 2025 Oct 30;21(9):3991–6. doi: 10.4103/NRR.NRR-D-25-00633 (PMC13557696; doi:10.4103/NRR.NRR-D-25-00633)
Supplement: Supplementary file 1 [file NRR-21-3991_Suppl1.pdf]

OPEN PEER REVIEW REPORT 1

Name of journal: Neural Regeneration Research
Manuscript NO: NRR-D-25-00633
Title: Lights for ageing: can photobiomodulation restore functionality in the cerebral networks of aged individuals?
Reviewer's Name: Dong-Wook Han
Reviewer's country: Republic of Korea

COMMENTS TO AUTHORS

Overall evaluation on article quality:

In the present work, the authors have explored the idea that the use of red and near infrared light - referred to often as photobiomodulation - can slow neuronal wear and tear, reduce the inflammation, enhance overall function of the cerebral networks and improve the quality of life in aged individuals. They hypothesized that the application of specific wavelengths of light has the potential to improve aged-induced cell dysfunction and death, leading to a restoration of more functional patterns of activity across the aged brain and reducing the risk of developing a neurological disorder such as stroke or neurodegenerative disease. The research seems an appropriate topic to be presented in this journal, but the author's scientific presentation needs further improvement.

Specific concerns:

1) The author has provided only schematic diagrams. Reproduced images from published articles can be incorporated at appropriate locations in the manuscript. I suggest including images illustrating the molecular pathways underlying neurodegenerative diseases, PBM stimulating mitochondrial enzymes, and the transcranial application of light.
2) In Figure 5, the term 'mitochondria' should be revised to 'mitochondrial function'.
3) For further understanding and deeper discussion, refer to this reference, doi.org/10.1016/j.biomaterials.2024.122623.
